# The Principles That Should Guide Us in the Rational Treatment of Gonorrhœa

**Published:** 1889-10

**Authors:** Bransford Lewis

**Affiliations:** St. Louis


### THE PRINCIPLES THAT SHOULD GUIDE US IN THE RATIONAL TREATMENT OF GONORRHŒA,

BY BRANSFORD LEWIS, M. D., ST. LOUIS.

If we were to reckon progress in medicine by stages, we could select no more apt a term for designating the present one than that of the Stage of Bacteriology. Bacteriology has assumed such an important *role* in all that relates to medicine or surgery, that our attention is drawn to it in the consideration of almost every subject in medical or surgical science. And yet, dropping from this lofty plane of thought, which tempts us into the field of glittering generalities, and limiting ourselves to the more practical question at hand, let us inquire what has this all-absorbing and comprehensive study and knowledge of bacteriology done for our assistance in the treatment of gonorrhœa? Has the hope of aborting, of quelling, of exterminating the disease by a treatment based on the discovery, study and acquaintance with the life history of that sturdy villain, the gonococcus, been realized? Have the methods of treatment thus brought into vogue accomplished the great wonders expected of them? Have they

* Read before the Mississippi Valley Medical Association, September, 1889.

accomplished anything more than the palliation, to a considerable extent, of the severity of the disease, of shortening, somewhat, the duration of its several stages, and of doing away with the old and barbarous forms of astringent, caustic and stimulating injections? I believe that all those who have tried this or that new antiseptic, this or that "infallible germicide," not simply on two or three isolated cases, in which beautiful results may have been attained, and which, by the way, were in all probability not gonorrhœa; I say, that all those who have tried such new antiseptic plans of treatment, in a considerable number of cases, are doubtless convinced that none are infallible; that all are subject to various influences, deleterious or favorable, that were met with in treating after the older, prudent methods.

Many beautiful and touching theories have been constructed to explain how the gonococcus would quail with fear when, in the midst of his revels, he should detect from afar the fumes of death-dealing iodoform brought into action by means of this or that preparation; or of how he would shrink with horror at the prospect of being literally boiled alive by hot injections; or washed out into the cold, cold world by the relentless flood of a prolonged irrigation; or of being crushed in spirit, body and soul, by the continuous presence of a medicated gelatine bougie; or dried up into an Egyptian mummy of a coccus in the arid soil of a mildly astringent, antiseptic, non-irritating, magic-healing, absorbent powder! But experience with these agents would seem to indicate that the usual rule of the breeding of contempt by familiarity is not broken in this instance. Nay, more! That the festive gonococcus after a time appears to become sufficiently acclimated to enjoy his surroundings, for a time, at least. And this, notwithstanding the fact that antiseptics do kill gonococci, and with great certainty and facility when they are in culture-fluids. But why not when they are in the urethra as well? For this reason: the gonococcus in preparing himself for the conflict does not foolishly remain where his foes can get at him with these various medicaments; he makes his landing and starts immediately for the woods, so to speak. He pushes on, by proliferation, between the epithelial cells, breaking through their connecting substance and finally ensconces himself below its deepest layers, along on the basement membrane, and even sometimes within and between the interlacing fibres of this structure. Here he proliferates and disseminates to his heart's content.

This has all been repeatedly and absolutely proved by eminent investigators; Bumm has watched the invasion of the conjunctival tissues by the hordes of gonococci; has seen them penetrate to the connective-tissue layer, and has noted the strong obstruction offered by this structure to their further progress. Not only this, but he has seen that the effect of astringents applied over the epithelial surface serves only to constringe and harden this covering, which then, indeed, forms a secure protection against the absorption or leaking through of any germicide or antiseptic which, embodied in the injection or what not, has been applied to the mucous surface. Moreover, he has seen that the elimination of the cocci contained in this meshwork of cells and fibres is brought about, not by the penetration of the germicides into the tissues, there to attack the organisms in their strongholds, as has been thought by some, but it is accomplished by a process of proliferation of the connective tissue fibres into which gonococci are unable to penetrate, as intimated above. In the stage of improvement, these fibres, incited by the irritation present, increase in number, push forward, driving before them the microbes towards the surface of the membrane, from which they are washed by the outgoing urine, or killed by the germicides. When a sufficiently strong connective-tissue bulwark has been constructed, new epithelial cells begin to dot the denuded surface here and there. The cocci have by this time lost much of their vitality and are unable to break them down with the ease shown at the first onslaught. The conditions for resisting their inroads, too, are then more perfect. They lie simply along the surface or among the superficial cells.

So that the final process of cure depends not altogether on the extermination of the few remaining gonococci, but also, and perhaps even more especially, on the closing of the tissues against their further invasion by the development of firm layers of this protecting barrier. And the inflammation then persisting may be interpreted as denoting the chronic irritation remaining after the severe disorganization wrought by the previous disease.

It is for these various reasons, then, that in the earlier stages when the gonococci themselves are doing the damage, the efficacy of antiseptics, germicides, astringents, etc., is limited to the position which they now occupy.

In order to overcome these impediments and give access of the medicines to the ambushed cocci, an enthusiastic Frenchman has recently suggested that the epithelial coat of the mucous membrane be scraped off by a brush-swab, on the plan commonly used in cleaning a pistol barrel; after which the urethra is to be douched with a powerful antiseptic solution. This method is original and ought to prove effective—in producing a stricture, if nothing else. It is certainly more energetic than any I should care to undertake.

I would therefore submit that efforts at aborting or killing a gonorrhœa with strong medicines, antiseptic or otherwise, not only do not attain the desired end, but are ill-advised and liable to be followed by unfortunate sequelæ or complications. Consequently, treatment should be based on a plan having for its object the idea of carrying the disease through its various stages, as authors used to say, *tuto, cito et jucunde;* allowing the patient to experience as little discomfort, pain and annoyance as possible; mollifying the inflammatory reaction and destroying, devitalizing and discouraging the gonococci as much as our rather restricted powers will admit of, and hastening the healing process with all possible speed.

To accomplish these ends, having used various forms and modes of treatment, I have concluded that the one offering, with the general run of cases, the most advantages with the fewest objections, is that of giving, in the first stage of the affection, simply alkaline dilutents and sedatives internally, making use of such adjuvants as dipping the penis in hot water, etc., and in the second and third stages giving injections of lanolin, medicated with an absolutely unirritating antiseptic, to which may be added in the third stage a mildly astringent and stimulating antiseptic.

As a means of introducing the ointment, I have been using, during the last six or seven months, this hard-rubber applicator which I present for your inspection. As you see, it consists of a catheter-like stem, perforated at its end, which is inserted into the urethra to the desired depth ; a box to contain the ointment, and a piston which is screwed into the box, driving the ointment before it into the stem and thence through the perforations into the urethra. When properly performed, an injection given with this instrument causes absolutely no pain or discomfort for the patient. But sometimes a sudden movement on his part will jog the stem against some tender spot and evoke an immediate and earnest protest. To obviate this, and to leave nothing undone that could in any way assist in avoiding irritation of any kind, I have had some vulcanized, soft rubber stems constructed, which answer the purpose very well. The square shape of the second (modified) box is of advantage in affording a securer hold on it. The stem need not be inserted as deep as its length will permit; the flexibility of the lanolin and elasticity of the urethral walls assure the spreading of the ointment over the inflamed area. Messrs. Aloe & Co., of St. Louis, have kindly constructed the instruments for me.

As to my reasons for preferring lanolin to other vehicles, I would say that, with reference to the other vehicles, water, the most common, is itself, in its purest state; irritating, and unless it contains some local anæsthetic will cause pain; powders or tablets, though dry and absorbent when first deposited, soon become moist and cake up, losing the properties for which they were chosen. Gelatine bougies give pain at every movement of the body until they are liquified; mucilages or emulsions present no advantages which are not possessed to a greater degree by lanolin, and—a point of great importance—all of them are lacking in "staying" qualities; with the first passage of urine out they go, and, in order to make their effect continuous, they must be renewed several times a day, entailing frequent repetition of the trouble, pain, etc., experienced each time.

Lanolin presents none of these disadvantages; it is wholly unirritating—is even soothing to inflamed tissues. When introduced pure, even without any pacifying sedatives, it invariably causes a feeling of relief and comfort to the patient who has been constantly reminded of his ailment by the teasing, harassing sensation incident to all cases of gonorrhœa. As one patient remarked, the ease afforded allowed him to forget all about it for hours at a time, whereas, before he began to receive the treatment, it was never out of his mind while he was awake. I think that the principal reason for this is, that it keeps the inflamed surfaces apart, preventing their continuous friction and auto-irritation. Actual pain in the urethra is also mollified by it.

The oiliness of lanolin assures its adhesion to the canal-walls, even in spite of the flushing of the urethra by the stream of urine. It may be noticed floating on the urine of the second or third passage after the application. It is evident that in this respect, too, it surpasses all of the excipients named. An authority tells us that lanolin possesses antiseptic properties of no mean order. I shall take up no more time in detailing its many advantages, which are almost self-evident.

As to the medicament employed, any remedy given in solution may be prescribed with equal propriety in lanolin. Of the various drugs which I have used, I sum up my impressions as follows: Bichloride of mercury, even in minute quantities, is too painful or irritating, and frequently causes an increase in the pus-formation; carbolic acid is also irritating but not painful; iodoform might be used were it less perniciously active in its odoriferousness. The zinc preparations are applicable to the later stages, in which they give material assistance towards shortening the wind-up. Resorcin would be a most admirable remedy were it not a most aggravating unstable drug. If administered after it has degenerated, it will not be long ere the operator has cause to regret his efforts in the way of economy. Boric acid directly following the increasing stage of the affection, seems to fulfill every indication; it is an antiseptic, a germicide, and yet has absolutely no irritating effect on the inflamed membrane. It is capable of killing the gonococci that it reaches and of preventing attacks from other microbes which give rise to the secondary, or mixed, infection of Bumm. And, by the way, the continuous presence of the medicated lanolin forms a vigilant guard against this complication.

Agreeing then, with the dictum of all authorities of the present day, that gonorrhœa is a specific disease which cannot be aborted after it is once fairly started, I conclude that

1st. Our treatment should not have for its object the futile idea of jugulating the disease in its early but established stages.

2d. The endeavor to control its severity, to lighten in every possible way all of its disagreeable features, to shorten its course and to ward off complications should be our guiding principles.

3d. No local agent does its share in fulfilling these indications more perfectly than does lanolin, medicated after the manner suggested.

*1006 Olive Street.*

